# Comparison of statistical approaches to predicting norovirus laboratory reports before and during COVID-19: insights to inform public health surveillance

**DOI:** 10.1038/s41598-023-48069-6

**Published:** 2023-12-05

**Authors:** Nikola Ondrikova, Helen Clough, Amy Douglas, Roberto Vivancos, Miren Itturiza-Gomara, Nigel Cunliffe, John P. Harris

**Affiliations:** 1 Institute of Infection, Veterinary and Ecological Sciences, University of Liverpool, Liverpool, UK; 2 Institute for Risk and Uncertainty, University of Liverpool, Liverpool, UK; 3 NIHR Health Protection Research Unit in Gastrointestinal Infections, University of Liverpool, Liverpool, UK; 4 National Surveillance Gastrointestinal Pathogens Unit, UK Health Security Agency, London, UK; 5 Health Protection Operations, UK Health Security Agency, Liverpool, UK; 6 NIHR Health Protection Research Unit in Emerging and Zoonotic Infections, University of Liverpool, Liverpool, UK; 7 Centre for Vaccine Innovation and Access, PATH, Geneva, Switzerland

**Keywords:** Infectious diseases, Statistics

## Abstract

Social distancing interrupted transmission patterns of contact-driven infectious agents such as norovirus during the Covid-19 pandemic. Since routine surveillance of norovirus was additionally disrupted during the pandemic, traditional naïve forecasts that rely only on past public health surveillance data may not reliably represent norovirus activity. This study investigates the use of statistical modelling to predict the number of norovirus laboratory reports in England 4-weeks ahead of time before and during Covid-19 pandemic thus providing insights to inform existing practices in norovirus surveillance in England. We compare the predictive performance from three forecasting approaches that assume different underlying structure of the norovirus data and utilized various external data sources including mobility, air temperature and relative internet searches (Time Series and Regularized Generalized Linear Model, and Quantile Regression Forest). The performance of each approach was evaluated using multiple metrics, including a relative prediction error against the traditional naive forecast of a five-season mean. Our data suggest that all three forecasting approaches improve predictive performance over the naïve forecasts, especially in the 2020/21 season (30–45% relative improvement) when the number of norovirus reports reduced. The improvement ranged from 7 to 22% before the pandemic. However, performance varied: regularized regression incorporating internet searches showed the best forecasting score pre-pandemic and the time series approach achieved the best results post pandemic onset without external data. Overall, our results demonstrate that there is a significant value for public health in considering the adoption of more sophisticated forecasting tools, moving beyond traditional naïve methods, and utilizing available software to enhance the precision and timeliness of norovirus surveillance in England.

## Introduction

In recent years, research on forecasting of infectious diseases and use of externally sourced data has grown considerably^1–3^. This has boosted the development of new modelling methods specifically targeting the challenges of using public health surveillance data^4^. In terms of external data sets, two popular choices across countries and pathogens are weather data such as air temperature, and digital data such as relative internet searches^5,6^. The importance of forecasting applications and use of externally sourced data in public health was further underlined by the Covid-19 pandemic. For example, mobility data played an important role in understanding the impact of Covid-19 prevention and control measures on human behaviour^7^. The transmission patterns and seasonality of pathogens unrelated to the cause of the pandemic such as those causing gastrointestinal infections were altered by the pandemic^8^. Additionally, temporal disruption to pathogen reporting hindered interpretation of the historical data that are used for comparison with an ongoing season to highlight changes in activity^9^.

Norovirus is a highly infectious viral agent causing short but intense symptoms of diarrhoea and vomiting^10^. The majority of reported norovirus infections in England originate from outbreaks in closed and semi-closed settings such as care homes and hospitals^10,11^. However, the surveillance system underestimates sporadic cases of norovirus infections, with 239 to 346 community cases for every laboratory-confirmed report^12^. The number of laboratory-confirmed cases of norovirus significantly decreased during the pandemic and this impact was more pronounced compared with a less outbreak-driven pathogen^8,9^. Previous research highlighted the importance of contact patterns and person-to-person transmission in the context of norovirus prediction^13^.

Currently, unusual activity of norovirus in England is highlighted by comparing the latest activity to historical data. Multiple sources of activity are available such as local outbreak reporting and laboratory-confirmed reporting. Additionally, the UK Health Security Agency (UKHSA) monitors circulating norovirus strains and captures real-time levels of gastrointestinal symptoms via syndromic surveillance. However, potential drawbacks of these approaches include (1) norovirus-specific reporting and strain monitoring has a built-in delay due to the time necessary to process stool samples; and (2) real-time syndromic surveillance inevitably contains false signals since it measures symptoms which can be attributed to gastrointestinal pathogens other than norovirus.

Externally sourced data have been used for norovirus prediction^14,15^, but more comprehensive evaluation of norovirus forecasting in England is lacking. This study investigates the predictive performance of three forecasting approaches with various covariates compared with the currently used naïve forecast of a week-based five-season mean before and during the Covid-19 pandemic. The three approaches cover popular choices in terms of family of methods (e.g. time series, regularized regression, algorithms) and data sources such as air temperature data and relative internet searches. Additionally, we test the statistical significance of the predictive performance improvement that can be achieved by the inclusion of Central England Temperature, Google Trends and Community Mobility data.

## Methods

### Data and pre-processing

#### Norovirus laboratory reports

We utilized weekly laboratory reports of norovirus infections that are routinely collected in England. The national data between Week 27, 2009 to Week 26, 2021 were extracted from the Second-Generation Surveillance System^16^. Data spans from summer (week 27) to summer (week 26) since norovirus reporting reaches peak activity in winter. While this data set is already curated, we added a zero entry in case no reports were received on a given week. The laboratory data are characterized as (1) non-negative integers, the minimum number of reports is zero, (2) autoregressive, current number of reports depends on the reports from previous weeks, (3) seasonal, visible peak in winter, (4) heteroskedastic, predictive error tends to be higher during the winter peak of norovirus activity, and (5) overdispersed, variance in the data is higher than expected.

Additionally, further categorical indicators were derived from the data based on exploratory analysis. Specifically, we indicate the week of Christmas holidays (Week 52), the initial weeks of the pandemic in England and the ongoing impact of the pandemic. We also used the laboratory data to fit norovirus seasonality with Fourier terms and calculate a three-week moving mean. As the laboratory data are inherently delayed due to the time required for a sample to reach diagnostic laboratories and to be tested and reported, it is common practice to not use the number of reports from the previous week. The smoothing via three-week moving mean allows leveraging of the available, but uncertain data point and increase of its reliability by combining with two datapoints prior.

#### Central England temperature

Central England Temperature (CET) is a time series indicating air temperature in England and is provided by Hadley Centre under the Met Office, a national meteorological service for the UK. Previous research suggests the Central England Temperature has a strong relationship with norovirus laboratory data^14^, but the variable has not been evaluated in the predictive modelling setting. We downloaded the daily data from the Met Office website^17^. Daily time series were aggregated to weekly level by calculating the mean value using “Zoo” R package^18^ and lagged (i.e. delayed) by three weeks to match the real-world setting where norovirus laboratory data lagged by one or two weeks are not available.

#### Community mobility data

Community Mobility data is a time series starting from 17^th^ February 2020. It captures the change in the number of visitors to public spaces in the time of the Covid-19 pandemic. Six categories of public spaces are available: Residential, Grocery and Pharmacy Stores, Retail and Recreation, Transit Stations and Workplaces. Due to a strong seasonal pattern in park visits, we excluded this category from the analysis. Note that the data captures only users who enabled sharing of their location history with Google.

**Figure 1:**
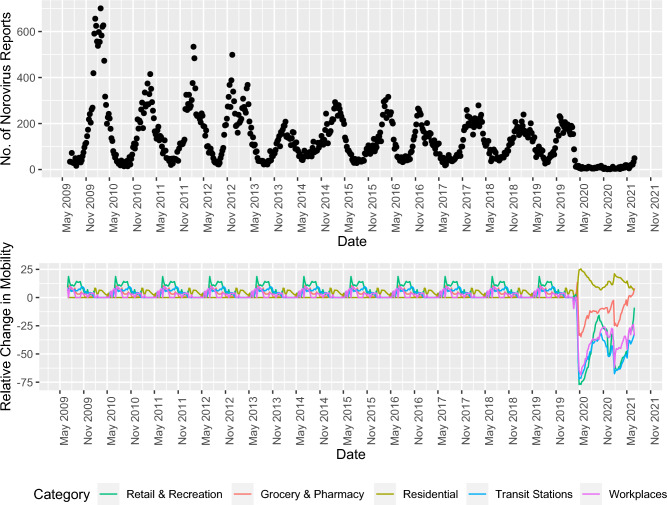
Norovirus laboratory reports and imputed Community Mobility Data.

Data can be extracted from Covid-19 Community Mobility Reports Google provides in pdf format. We used a pre-processed and smoothed version of the time series from Our World in Data^19^ downloadable in a csv format and better suited for dynamic analysis. Similar to the CET time series, mobility data were adjusted to weekly granularity by calculating a mean across daily values and lagged by three weeks. Additionally, further experimentation was necessary to determine handling of the missing data points before February 17, 2020. We considered two alternatives, (1) replacing missing data with random sequence based on the random noise component from the individual time series between June 2021 and June 2022, and (2) extracting seasonal component and the mean of the random noise component. To extract seasonality and random noise, each mobility time series was decomposed using a decompose function from the “stats” R package^20^. The two options were compared in the Count Time Series GLM model described later. The lower AIC indicated the option with the seasonal component (AIC = 5758.79) filled the missing values better in comparison to the option without seasonal component (AIC = 6104.98). Figure 1 shows the mobility data time series alongside norovirus laboratory reports.

#### Relative search volumes

For relative internet searches we used public application programming interface (API) for R implemented in “gtrendsR” package^21^, which allowed us to access and download Google Trends results programmatically. Google trends offer relative internet search volumes, which means that selecting a different period can result in different values for a particular week. Therefore, to faithfully represent the real-world setting, the data for the prediction assessment were extracted week-by-week, i.e. the data is downloaded as a fresh set of data every week when predictions are produced.

Search term selection was conducted in an automated manner described here^22^. Briefly, we downloaded norovirus-related search terms by first extracting internet searches for the keywords “norovirus” and “stomach bug” and continuing with all the related search terms provided by Google Trends. We then repeated the same process for all the related search terms. Finally, the time series for the identified search terms were lagged by three weeks, merged with the national norovirus laboratory reports and Pearson’s correlation coefficient was calculated. Search terms including a number and those weakly correlated with laboratory report (coefficient < 0.20) were removed. The selection period ranged from September 2014 and 2019 and resulted in 14 search terms: "symptoms norovirus", "norovirus how long", "symptoms of norovirus", "norovirus incubation", "norovirus treatment", "what is norovirus", "norovirus outbreak", "sickness bug", "stomach bug", "norovirus first symptoms", "norovirus", "stomach flu", "flu symptoms" and "gastric flu".

### Statistical analysis

The goal of the statistical analysis is to predict the number of norovirus laboratory reports 4 weeks ahead following the last report in the time of pandemic. We selected three approaches for comparison based on the statistical characteristics of the laboratory data and the ability to incorporate additional data sources—generalized linear model (GLM) for count time series, Regularized GLM with Cross Validation and Quantile Regression Forest. The response variable is the same in all the approaches—the weekly time series of norovirus reports in England. However, not all the approaches use all of the data sources or newly created explanatory variables described in the Data and Pre-processing section. This is summarized in Table 1.

**Table 1:** List of variables and modelling approaches.

List of variables	Variable type	Modelling approaches		
		Time series GLM	Regularized GLM	Quantile regression forest
Count of norovirus laboratory reports	Outcome/target	✓	✓	✓
3-week rolling mean	Covariate/feature		✓	✓
Seasonal pattern fitted with Fourier terms	Covariate/feature		✓	✓
Central England Temperature	Covariate/feature	✓	✓	✓
Google Trends	Covariate/feature	✓	✓	✓
Google Community Mobility	Covariate/feature	✓	✓	✓
Categorical indicators:				
Christmas (week 52 every year)	Covariate/feature	✓		
Early Covid-19 pandemic (weeks 12 to 26, 2020)	Covariate/feature	✓		
Covid-19 pandemic (week 12, 2020–week 26, 2021)	Covariate/feature	✓		

After the data is imported and prepared for the analysis, we split the merged data set into two sections: training and testing. To estimate the error, we dynamically extend the training data in a stepwise fashion, week-by-week. Testing data are then the 4 following data points. This means that the training iteration always started in Week 27, 2009, but ended in different weeks. For example, the first iteration of the training data ended in Week 38, 2017, the second in Week 39, 2017 and the last iteration ended in Week 19, 2021. Equivalent testing data ranges were Weeks 39–42, 2017 for the first evaluation iteration, Weeks 40–43, 2017 for the second and Weeks 20–23, 2021 for the last. We only evaluated the period between calendar weeks 40 to 20 which is more difficult to predict as it includes the high season due to outbreaks. Note the difference between the calendar weeks and the training/testing data weeks. In the real-world setting, the predictions generated on the 40^th^ calendar week, could only use data up-until week 38 due to reporting delay and so the 1-week-ahead forecast is a hindcast, 2-weeks-ahead is a nowcast and the 3 and 4-week-ahead predictions are true forecasts. During this procedure, we kept track of the prediction type (1-week-ahead, 2-weeks-ahead, etc.) and the norovirus season (e.g. Week 27, 2017–Week 26, 2018).

Finally, predictions from the testing data are evaluated against the observed norovirus laboratory reports with two metrics: weighted interval score (WIS)^23^ implemented in “scoringUtils” package^24^ and Pearson Correlation Coefficient from “stats” package^20^ . WIS assess the forecasting approaches based on prediction intervals and the lower the score the better. Pearson coefficient indicates trend of point predictions and higher values indicate better trend prediction. The results are presented for each of the four prediction types. The result section also provides pre-pandemic, early pandemic and post-pandemic onset aggregations with and without the inclusion of a particular externally sourced data. A permutation test is used to assess statistical difference between the WIS metric across the pre-, early and post-pandemic onset aggregations with and without CET, Google Trends and mobility data within each modelling framework.

To compare modelling forecasts to the naïve forecast, we use relative Mean Absolute Error (rMAE) that assess point-prediction accuracy. Naïve forecasts are calculated as a mean of historical values across previous 5 years for a given week, i.e. five-season mean. For example, five-season mean for a week 40, 2020 would be calculated as a mean of week 40 from years 2019, 2018, 2017, 2016 and 2015.

### Generalized linear model for count time series

Generalized Linear Model (GLM) for count time series is a simplified name for Integer-valued Generalized Autoregressive Conditional Heteroskedasticity (IN-GARCH)^25^ models. These models are based on generalized linear regression (GLM) utilising quasi conditional maximum likelihood estimation to fit a negative binomial distribution that can account for overdispersion. The method is implemented in R in the “tscount” package^26^. The main advantage of this implementation is the convenience of straight forward specification. We can consider the seasonal and autoregressive nature of the norovirus laboratory reports by regressing on past values. Therefore, we did not use pre-fitted Fourier terms seasonality and three-week moving mean as explanatory variables. However, CET, the community mobility data, linear trend and categorical indicators for Christmas and pandemic were included.

### Regularized GLM with cross-validation

Regularisation is a commonly used method to extract a signal from a high number of explanatory variables as it shrinks coefficients of less relevant covariates. The regularization parameter is selected through a process of cross-validation. The parameter achieving the lowest prediction error on the validation/testing portion of the data is selected for the regularization. The implementation from the “mpath” R package^27^ fits a negative binomial model via penalized maximum likelihood where the penalized log-likelihood is maximized. We used this method to extract the relevant signal from relative search volumes from Google Trends, mobility and air temperature data to predict the number of norovirus laboratory reports. As this method does not assume a sequential nature of the data, we included a 3-week rolling mean and pre-fitted seasonality derived from norovirus laboratory reports to account for autocorrelation and seasonality. Categorical variables were excluded as this is not a time-series model. Prediction intervals were derived from a GLM following Negative Binomial distribution without regularization implemented in “MASS” package^28^.

### Quantile regression forest

Quantile regression forest is a nonlinear method that does not assume a specific data distribution. As the random forest, at its core is a high number of binary decision trees built from bootstrapped data samples. Individual predictions from the trees are then aggregated. Additionally, the trees choose random predictors at every split. Quantile regression forest is a generalisation of random forest. The main advantage is that it provides approximations of the full conditional distribution compared with a mean value provided by random forest. We used the implementation of the method in “quantregForest” R package^29^. The explanatory variables used in this model include CET and mobility data. Similarly, to Regularized regression, categorical variables were excluded and autocorrelation and seasonality were handled by pre-fitted seasonality and 3-week moving mean.

## Results

In this section, forecasting performance of each approach is presented for each forecasting horizon and model type based on which external dataset was included in a stepwise fashion. We consider three periods, the first norovirus season during the pandemic (week 40, 2020–week 20, 2021), the early Covid-19 pandemic and end of norovirus season 2019–2020 (week 12–week 20, 2020) and the pre-pandemic period (week 40, 2017–week 11, 2020). We tested the differences between models within forecasting approaches weighted interval scores for all period using permutation test.

**Figure 2:**
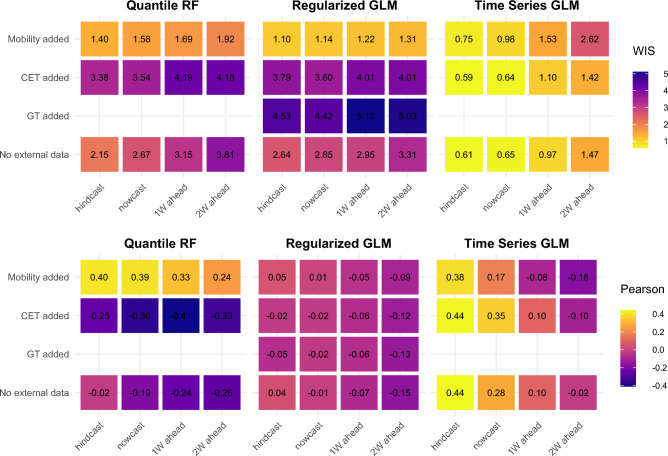
Comparison of predictive performance across forecasting approaches: the first norovirus season during the Covid-19 pandemic (week 40, 2020–week 20, 2021).

Comparison of predictive performance during the 2020/21 norovirus season (Fig. 2) indicates that including Google Community Mobility data significantly improved the WIS of Quantile Regression Forest (p < 0.001) and Negative Binomial GLM (p < 0.010) compared to model versions with no external data. Count Time Series GLM did not benefit from inclusion of mobility or CET data during this period (Supplementary Table 1). In terms of trend prediction, QRF with mobility data showed the strongest coefficient across predictive horizon (0.40 > ρ > 0.24). Regularized Negative Binomial GLM showed the weakest correlation.

**Figure 3:**
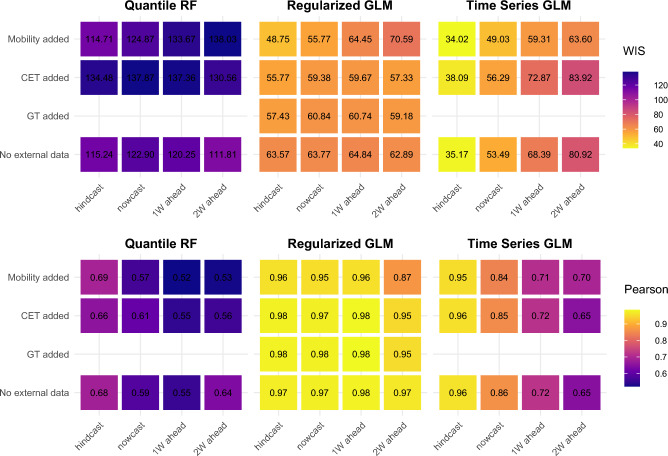
Comparison of predictive performance across forecasting approaches: early Covid-19 pandemic period and the end of norovirus season 2019–2020 (week 12–week 20, 2020).

In contrast, the correlations of predicted and observed values are the strongest for the Regularized GLM during the early period of the pandemic, while QRF shows the weakest association (Fig. 3). This pattern is present in the weighted interval scores too. Regularized GLM models with CET and Google trends data provide significantly lower WIS for the 1–2 week forecasts (p < 0.009) and the inclusion of mobility data provides similar performance to the baseline Regularized GLM. However, Time series GLM nowcast and 1–2 week forecasts benefit from CET and mobility data significantly (p < 0.045).

**Figure 4:**
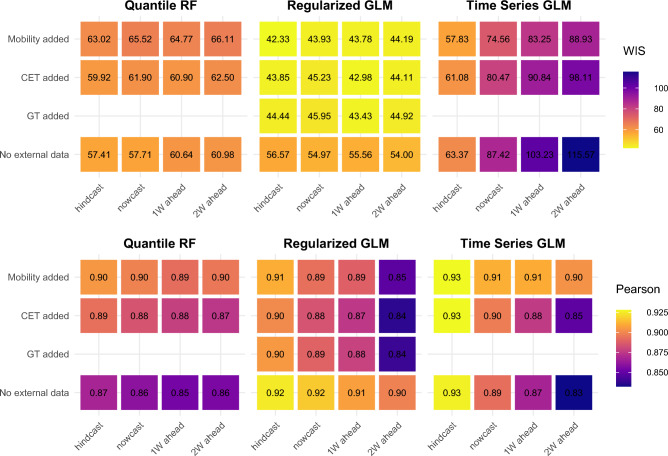
Comparison of predictive performance across forecasting approaches: Covid-19 pre-pandemic period (week 40, 2017–week 11, 2020).

Even in the pre-pandemic period (Fig. 4), seasonal components of the mobility variables improve the accuracy and showed stronger correlation for the Time Series GLM-based approach, particularly for the future horizon (3–4 weeks ahead). Additionally, the weighted interval score improved significantly when CET and mobility seasonal component were added for all predictive horizons (p = 0.000). Concerning Regularized GLM approach, WIS also improves with every addition of external data sets significantly (p = 0.000) compared to the model without externally sourced data. On the other hand, adding external data to the QRF approach does not provide significantly different predictive performance compared to the basic model.

Inclusion of CET and seasonal component of mobility variables on top of Google Trends, shows only a small reduction in WIS (Fig. 4). In contrast, the trend prediction is impacted negatively when externally sourced data are integrated into the Regularized GLM model.

Finally, we compare all point-predictions from the forecasting models to the naïve forecast in the form of five-season mean (Supplementary Table 2). All models tend to regress to the historical mean as the time passes, i.e. the 4-weeks ahead forecasting MAE tends to be closer to the five-season MAE. During 2020/21 norovirus season, models with mobility data showed lower MAE by 45% (QRF), 35% (Regularized GLM), 30% (Time Series GLM) on average across forecasting horizons. However, the improvement of point-prediction accuracy against five-season mean is lower for the 4-weeks ahead forecast across models and approaches during the same period—0–30% (QRF), 0–31% (Regularized GLM), 0–8% (Time Series GLM).

Relative MAE for the pre-pandemic period shows that approaches with seasonal components of mobility data improve the point accuracy compare to the five-season mean by 7% (QRF), 22% (Regularized GLM), 17% (Time Series GLM) on average across forecasting horizons.

## Discussion

Predictions of confirmed norovirus infections can provide a timely indicator of current and near-future norovirus activity. Our study underscores the utility of applying statistical modelling and integrating externally sourced data into norovirus surveillance, especially in the context of the COVID-19 pandemic. By comparing three distinct statistical approaches, we demonstrated that each method outperformed the naïve forecast, traditionally used in norovirus surveillance in England. Our results highlight the need for adaptable forecasting models. The relative point-prediction improvement of our models reached 30–45% compared to the naïve forecast during the 2020/21 norovirus season which was impacted by the pandemic. This is expected considering the naïve forecast is based on historical seasonal mean, even if reduced by 90% in scale. However, we demonstrated that forecasting approaches explored in this study can improve the point-prediction between 7 and 22% on average across the 4-week forecasting horizons even before the pandemic. This suggests, that with norovirus laboratory reports returning to their previous seasonal pattern, statistical models from this study are likely to provide point-prediction improvement over the historical seasonal mean after the Covid-19 pandemic.

The results focused on the first norovirus season following the Covid-19 pandemic indicate that the Count Time Series GLM approach provides the best performance when no external data is used. Its predictive scores were very similar to those from Regularized GLM incorporating air temperature, internet searches and mobility data. However, the Time Series GLM approach showed improved predictions when Community Mobility variables were included as covariates early in the pandemic. Further, our findings show that even the backfilled mobility data based on seasonal component improved predictive performance of GLM-based models during the pre-pandemic period when the real-time data was not available. Quantile Regression Forest showed the poorest predictive performance for the period of early pandemic suggesting this approach is not sensitive to sudden changes in the data. Including mobility variables in QRF in the first norovirus season after the pandemic onset improved the forecasting scores and predicted the trend better than the other two approaches.

In the period before the Covid-19 pandemic, Regularized GLM with Negative Binomial distribution showed the best predictive performance in terms of forecasting score and showed 35% improvement in the point-prediction when compared to the naïve forecast (five-season mean). While the performance improved when we included the Google Trends variables as covariates, the improvement became incremental with inclusion of further variables—Central England Temperature, seasonal component of Mobility data. Conversely, trend prediction deteriorated when external data sources were incorporated. Therefore, it is important to consider the specific practical application of a forecasting model. Given the challenges of acquiring and selecting relevant Google Trends data, the effort required in some instances might outweigh the benefits for enhancing norovirus forecasting in England.

The improvement in predictive performance through mobility data underlines the importance of societal, economic and cultural trends for predicting highly contagious pathogens. Decreased mixing in public places can have a long-term impact on infections driven by contact patterns, including decreased levels of norovirus infections. Early in the Covid-19 pandemic, norovirus laboratory reports decreased with reduced mobility, likely due to genuinely decreased transmission^8,9^. As Covid-19 control measures were relaxed, exploratory analysis of the mobility data still suggests a shift in the visit frequencies to workplaces, groceries, pharmacies, etc. People spend 5% more time in residential areas and less in transport stations and workplaces compared with before the pandemic. A report from McKinsey Global institute suggests that the remote working trend might slow down but persist in advanced economies^30^. Thus, we can expect the changes in levels of mixing to continue and consequently impact norovirus transmission. Additionally, the remote work trend concerns mainly white-collar work, which has the potential to further exacerbate existing health inequalities regarding gastrointestinal infections^31^.

Previous studies showed that short-term forecasting successfully delivered more accurate predictions than traditional and easy-to-obtain naive forecasts such as mean across the past seasons for a given week^4,32^. However, the accuracy of long-term forecasts was similar to the naïve forecasts. We also observed this effect with all approaches. Therefore, it is essential to differentiate between application contexts and whether long- or short-term forecasting can bring most benefits. Naïve forecasts such as the five-season mean remain better suited in contexts where long-term disease activity pattern needs to be considered. However, the relatively more accurate short-term predictions of highly contagious infection agents such as norovirus can be helpful in weekly or monthly capacity planning at hospitals. Also, it is important to note the difference between explanatory or descriptive modelling and predictive modelling^33^. Long -term contexts may still utilize statistical modelling to improve understanding of the underlaying process generating surveillance data but in the short-term, when the predictive performance of the models is the goal, machine learning approaches can be more suitable^34^. Our results demonstrate that the predictive performance of the Quantile Regression Forest and Regularized GLM benefited from external data more than the time series approach during the first norovirus season after the Covid-19 pandemic onset. On the other hand, Time Series GLM allowed for time dependent categorical indicators to be specified such as weeks of the Covid-19 pandemic onset and so no external data were required to achieve good predictive performance.

Developing a process to estimate pathogen activity that relies on externally produced data carries risks from the public health perspective. Thus, the effort required to adapt a modelling approach and pre-process the external data would be in vain if the external data set became unavailable. This is exemplified with the community mobility data when in October 2022, Google stopped producing new reports. However, the results show that our approximation of respective Community Mobility variables improve the prediction of norovirus laboratory reports from the Time Series GLM in the period before the Covid-19 pandemic. This suggests that the combination of seasonality and noise derived from the available data could be used to fill in missing time points, serving as a continuation of the real-time data in upcoming seasons. In this manner, we can account for the disruption in norovirus activity during the pandemic while also addressing the absence of data after it became unavailable. Nonetheless, further research is needed to confirm the effectiveness of this approach. Additionally, a variety of private mobility data sources similar to the one used in this study can be accessed via Development Data Partnership Organisation^35^. Considering the improvements achieved by using Community Mobility data from Google specifically, this data set should be included in the Development Data Partnership for public health benefit.

In terms of limitations, the forecasting approach leveraging Google Trends could be further refined if the search-term selection is repeated at the beginning of every season, since the specific terms can change as language and public familiarity with a particular pathogen change^22^. Additionally, the study was undertaken before norovirus laboratory report counts had rebounded to pre-pandemic levels. Nonetheless, recent data from the 2022/23 season^36^ imply a return to previous norovirus activity patterns, suggesting the applicability of insights gained from pre-pandemic and pandemic models. Moreover, the study highlights that existing software offers good options for norovirus forecasting that can supplement the naïve forecast currently used in norovirus surveillance practice. Future research could focus on understanding what methods predicted the rebound in norovirus laboratory reports most accurately.

In conclusion, this study describes the potential of externally sourced data to improve the accuracy of norovirus forecasting in England. The results demonstrate that incorporating mobility data and derived data signals can significantly improve the accuracy of some forecasting models, particularly during the Covid-19 pandemic. However, different forecasting approaches forecast norovirus reports more accurately across investigated time periods. While Regularized GLM showed the best predictions before the pandemic, the Count Time Series GLM without any externally sourced data displayed the strongest forecasting score in the first norovirus season after the Covid-19 pandemic onset. Finally, our research suggests that there is significant value for public health in considering the adoption of more sophisticated forecasting tools, moving beyond traditional naïve methods, and utilizing available software to enhance the precision and timeliness of norovirus surveillance in England.

### Supplementary Information

Supplementary Tables.

## Data Availability

Routine surveillance data cannot be shared publicly because the provision of the data is dependent on the intended use. The R code and synthetic data are available at https://doi.org/10.5281/zenodo.10032003. Public API to acquire Google Trends and the Our World in Data website providing access to Community Mobility Data are referenced^19,21^.
