# Supplementary material for: Comparison of statistical approaches to predicting norovirus laboratory reports before and during COVID-19: insights to inform public health surveillance
Source: Sci Rep. 2023 Dec 5;13:21457. doi: 10.1038/s41598-023-48069-6 (PMC10697939; doi:10.1038/s41598-023-48069-6)
Supplement: Supplementary file 1 — Supplementary Tables. [file 41598_2023_48069_MOESM1_ESM.pdf]

Supplement

Table 1: Significance tests for differences between interval scores predictive errors of simplest versions of forecasting approaches and versions when different datasets were added.

**Prediction Type**	**Pandemic timeline**	**Model comparison against “No external data”**	**Forecasting approach**	**Observed score difference** e.g. basic – CET added	**Permutation Test: P-value**
**Hindcast**	Pre	CET added	Time Series GLM	2.29	0.000
**Hindcast**	Pre	Mobility added	Time Series GLM	5.54	0.000
**Nowcast**	Pre	CET added	Time Series GLM	6.95	0.000
**Nowcast**	Pre	Mobility added	Time Series GLM	12.86	0.000
**1W ahead**	Pre	CET added	Time Series GLM	12.40	0.000
**1W ahead**	Pre	Mobility added	Time Series GLM	19.98	0.000
**2W ahead**	Pre	CET added	Time Series GLM	17.46	0.000
**2W ahead**	Pre	Mobility added	Time Series GLM	26.64	0.000
**Hindcast**	Early	CET added	Time Series GLM	-2.92	0.006
**Hindcast**	Early	Mobility added	Time Series GLM	1.16	0.342
**Nowcast**	Early	CET added	Time Series GLM	-2.80	0.001
**Nowcast**	Early	Mobility added	Time Series GLM	4.46	0.045
**1W ahead**	Early	CET added	Time Series GLM	-4.48	0.000
**1W ahead**	Early	Mobility added	Time Series GLM	9.08	0.004
**2W ahead**	Early	CET added	Time Series GLM	-2.99	0.014
**2W ahead**	Early	Mobility added	Time Series GLM	17.33	0.003
**Hindcast**	Post	CET added	Time Series GLM	-1.17	0.005
**Hindcast**	Post	Mobility added	Time Series GLM	0.57	0.122
**Nowcast**	Post	CET added	Time Series GLM	-2.35	0.012
**Nowcast**	Post	Mobility added	Time Series GLM	1.33	0.088
**1W ahead**	Post	CET added	Time Series GLM	-2.91	0.070
**1W ahead**	Post	Mobility added	Time Series GLM	2.50	0.038
**2W ahead**	Post	CET added	Time Series GLM	-3.18	0.214
**2W ahead**	Post	Mobility added	Time Series GLM	3.26	0.056
**Hindcast**	Pre	CET added	Quantile Regression Forest	-2.51	0.407
**Hindcast**	Pre	Mobility added	Quantile Regression Forest	-5.60	0.154
**Nowcast**	Pre	CET added	Quantile Regression Forest	-4.19	0.168
**Nowcast**	Pre	Mobility added	Quantile Regression Forest	-7.81	0.044
**1W ahead**	Pre	CET added	Quantile Regression Forest	-0.26	0.935
**1W ahead**	Pre	Mobility added	Quantile Regression Forest	-4.13	0.358
**2W ahead**	Pre	CET added	Quantile Regression Forest	-1.52	0.637
**2W ahead**	Pre	Mobility added	Quantile Regression Forest	-5.13	0.282
**Hindcast**	Early	CET added	Quantile Regression Forest	-19.24	0.056
**Hindcast**	Early	Mobility added	Quantile Regression Forest	0.53	0.963
**Nowcast**	Early	CET added	Quantile Regression Forest	-14.97	0.161

**Nowcast**	Early	Mobility added	Quantile Regression Forest	-1.98	0.854
**1W ahead**	Early	CET added	Quantile Regression Forest	-17.11	0.107
**1W ahead**	Early	Mobility added	Quantile Regression Forest	-13.42	0.347
**2W ahead**	Early	CET added	Quantile Regression Forest	-18.75	0.074
**2W ahead**	Early	Mobility added	Quantile Regression Forest	-26.21	0.082
**Hindcast**	Post	CET added	Quantile Regression Forest	-51.57	0.000
**Hindcast**	Post	Mobility added	Quantile Regression Forest	40.53	0.000
**Nowcast**	Post	CET added	Quantile Regression Forest	-49.80	0.000
**Nowcast**	Post	Mobility added	Quantile Regression Forest	33.62	0.000
**1W ahead**	Post	CET added	Quantile Regression Forest	-55.28	0.000
**1W ahead**	Post	Mobility added	Quantile Regression Forest	29.79	0.000
**2W ahead**	Post	CET added	Quantile Regression Forest	-51.14	0.000
**2W ahead**	Post	Mobility added	Quantile Regression Forest	22.39	0.001
**Hindcast**	Pre	CET added	Regularized GLM	12.73	0.000
**Hindcast**	Pre	GT added	Regularized GLM	12.73	0.000
**Hindcast**	Pre	Mobility added	Regularized GLM	14.24	0.000
**Nowcast**	Pre	CET added	Regularized GLM	9.75	0.000
**Nowcast**	Pre	GT added	Regularized GLM	9.75	0.000
**Nowcast**	Pre	Mobility added	Regularized GLM	11.05	0.000
**1W ahead**	Pre	CET added	Regularized GLM	12.57	0.000
**1W ahead**	Pre	GT added	Regularized GLM	12.57	0.000
**1W ahead**	Pre	Mobility added	Regularized GLM	11.78	0.000
**2W ahead**	Pre	CET added	Regularized GLM	9.89	0.000
**2W ahead**	Pre	GT added	Regularized GLM	9.89	0.000
**2W ahead**	Pre	Mobility added	Regularized GLM	9.81	0.000
**Hindcast**	Early	CET added	Regularized GLM	7.80	0.016
**Hindcast**	Early	GT added	Regularized GLM	7.80	0.045
**Hindcast**	Early	Mobility added	Regularized GLM	14.82	0.005
**Nowcast**	Early	CET added	Regularized GLM	4.39	0.066
**Nowcast**	Early	GT added	Regularized GLM	4.39	0.154
**Nowcast**	Early	Mobility added	Regularized GLM	8.00	0.023
**1W ahead**	Early	CET added	Regularized GLM	5.17	0.008
**1W ahead**	Early	GT added	Regularized GLM	5.17	0.009
**1W ahead**	Early	Mobility added	Regularized GLM	0.39	0.974
**2W ahead**	Early	CET added	Regularized GLM	5.56	0.002
**2W ahead**	Early	GT added	Regularized GLM	5.56	0.006
**2W ahead**	Early	Mobility added	Regularized GLM	-7.70	0.446
**Hindcast**	Post	CET added	Regularized GLM	3.84	0.010
**Hindcast**	Post	GT added	Regularized GLM	3.84	0.070
**Hindcast**	Post	Mobility added	Regularized GLM	28.23	0.000
**Nowcast**	Post	CET added	Regularized GLM	6.24	0.000
**Nowcast**	Post	GT added	Regularized GLM	6.24	0.376
**Nowcast**	Post	Mobility added	Regularized GLM	29.79	0.000

**1W ahead**	Post	CET added	Regularized GLM	6.07	0.000
**1W ahead**	Post	GT added	Regularized GLM	6.07	0.154
**1W ahead**	Post	Mobility added	Regularized GLM	30.07	0.000
**2W ahead**	Post	CET added	Regularized GLM	6.71	0.000
**2W ahead**	Post	GT added	Regularized GLM	6.71	0.168
**2W ahead**	Post	Mobility added	Regularized GLM	30.77	0.000

Table 2: Relative Mean Absolute Score (rMAE) between all models and naïve forecast (5 season mean).

Prediction Type	Pandemic timeline	Model type	Forecasting approach	Model MAE	5-season mean MAE	Relative MAE
**Hindcast**	Pre	basic	Time Series GLM	18.40	25.24	0.73
**Hindcast**	Pre	CET added	Time Series GLM	18.38	25.24	0.73
**Hindcast**	Pre	Mobility added	Time Series GLM	18.69	25.24	0.74
**Nowcast**	Pre	basic	Time Series GLM	22.68	25.24	0.90
**Nowcast**	Pre	CET added	Time Series GLM	22.36	25.24	0.89
**Nowcast**	Pre	Mobility added	Time Series GLM	21.26	25.24	0.84
**1W ahead**	Pre	basic	Time Series GLM	25.38	25.24	1.01
**1W ahead**	Pre	CET added	Time Series GLM	24.65	25.24	0.98
**1W ahead**	Pre	Mobility added	Time Series GLM	21.46	25.24	0.85
**2W ahead**	Pre	basic	Time Series GLM	28.35	25.24	1.12
**2W ahead**	Pre	CET added	Time Series GLM	27.25	25.24	1.08
**2W ahead**	Pre	Mobility added	Time Series GLM	22.92	25.24	0.91
**Hindcast**	Early	basic	Time Series GLM	12.90	23.35	0.55
**Hindcast**	Early	CET added	Time Series GLM	13.94	23.35	0.60
**Hindcast**	Early	Mobility added	Time Series GLM	13.44	23.35	0.58
**Nowcast**	Early	basic	Time Series GLM	20.74	23.35	0.89
**Nowcast**	Early	CET added	Time Series GLM	22.15	23.35	0.95
**Nowcast**	Early	Mobility added	Time Series GLM	21.42	23.35	0.92
**1W ahead**	Early	basic	Time Series GLM	28.10	23.35	1.20
**1W ahead**	Early	CET added	Time Series GLM	30.78	23.35	1.32
**1W ahead**	Early	Mobility added	Time Series GLM	27.75	23.35	1.19
**2W ahead**	Early	basic	Time Series GLM	36.17	23.35	1.55
**2W ahead**	Early	CET added	Time Series GLM	39.46	23.35	1.69
**2W ahead**	Early	Mobility added	Time Series GLM	32.84	23.35	1.41
**Hindcast**	Post	basic	Time Series GLM	3.85	8.23	0.47
**Hindcast**	Post	CET added	Time Series GLM	4.06	8.23	0.49
**Hindcast**	Post	Mobility added	Time Series GLM	3.91	8.23	0.48

**Nowcast**	Post	basic	Time Series GLM	4.84	8.23	0.59
**Nowcast**	Post	CET added	Time Series GLM	4.90	8.23	0.60
**Nowcast**	Post	Mobility added	Time Series GLM	4.72	8.23	0.57
**1W ahead**	Post	basic	Time Series GLM	5.91	8.23	0.72
**1W ahead**	Post	CET added	Time Series GLM	6.04	8.23	0.73
**1W ahead**	Post	Mobility added	Time Series GLM	6.46	8.23	0.78
**2W ahead**	Post	basic	Time Series GLM	7.60	8.23	0.92
**2W ahead**	Post	CET added	Time Series GLM	7.91	8.23	0.96
**2W ahead**	Post	Mobility added	Time Series GLM	8.36	8.23	1.02
**Hindcast**	Pre	basic	Quantile Regression Forest	26.87	25.24	1.06
**Hindcast**	Pre	CET added	Quantile Regression Forest	24.00	25.24	0.95
**Hindcast**	Pre	Mobility added	Quantile Regression Forest	22.74	25.24	0.90
**Nowcast**	Pre	basic	Quantile Regression Forest	27.88	25.24	1.10
**Nowcast**	Pre	CET added	Quantile Regression Forest	24.95	25.24	0.99
**Nowcast**	Pre	Mobility added	Quantile Regression Forest	23.63	25.24	0.94
**1W ahead**	Pre	basic	Quantile Regression Forest	28.13	25.24	1.11
**1W ahead**	Pre	CET added	Quantile Regression Forest	25.79	25.24	1.02
**1W ahead**	Pre	Mobility added	Quantile Regression Forest	24.38	25.24	0.97
**2W ahead**	Pre	basic	Quantile Regression Forest	28.42	25.24	1.13
**2W ahead**	Pre	CET added	Quantile Regression Forest	25.81	25.24	1.02
**2W ahead**	Pre	Mobility added	Quantile Regression Forest	23.43	25.24	0.93
**Hindcast**	Early	basic	Quantile Regression Forest	48.87	23.35	2.09
**Hindcast**	Early	CET added	Quantile Regression Forest	56.80	23.35	2.43
**Hindcast**	Early	Mobility added	Quantile Regression Forest	45.67	23.35	1.96
**Nowcast**	Early	basic	Quantile Regression Forest	54.93	23.35	2.35
**Nowcast**	Early	CET added	Quantile Regression Forest	64.30	23.35	2.75
**Nowcast**	Early	Mobility added	Quantile Regression Forest	53.80	23.35	2.30
**1W ahead**	Early	basic	Quantile Regression Forest	55.93	23.35	2.40
**1W ahead**	Early	CET added	Quantile Regression Forest	68.07	23.35	2.92
**1W ahead**	Early	Mobility added	Quantile Regression Forest	61.13	23.35	2.62
**2W ahead**	Early	basic	Quantile Regression Forest	56.13	23.35	2.40
**2W ahead**	Early	CET added	Quantile Regression Forest	67.73	23.35	2.90
**2W ahead**	Early	Mobility added	Quantile Regression Forest	68.70	23.35	2.94
**Hindcast**	Post	basic	Quantile Regression Forest	10.15	8.23	1.23
**Hindcast**	Post	CET added	Quantile Regression Forest	20.05	8.23	2.44
**Hindcast**	Post	Mobility added	Quantile Regression Forest	3.62	8.23	0.44

**Nowcast**	Post	basic	Quantile Regression Forest	15.06	8.23	1.83
**Nowcast**	Post	CET added	Quantile Regression Forest	26.84	8.23	3.26
**Nowcast**	Post	Mobility added	Quantile Regression Forest	3.91	8.23	0.47
**1W ahead**	Post	basic	Quantile Regression Forest	17.61	8.23	2.14
**1W ahead**	Post	CET added	Quantile Regression Forest	31.83	8.23	3.87
**1W ahead**	Post	Mobility added	Quantile Regression Forest	4.63	8.23	0.56
**2W ahead**	Post	basic	Quantile Regression Forest	20.95	8.23	2.55
**2W ahead**	Post	CET added	Quantile Regression Forest	36.78	8.23	4.47
**2W ahead**	Post	Mobility added	Quantile Regression Forest	5.75	8.23	0.70
**Hindcast**	Pre	basic	Regularized GLM	18.27	25.24	0.72
**Hindcast**	Pre	GT added	Regularized GLM	19.14	25.24	0.76
**Hindcast**	Pre	CET added	Regularized GLM	19.44	25.24	0.77
**Hindcast**	Pre	Mobility added	Regularized GLM	18.32	25.24	0.73
**Nowcast**	Pre	basic	Regularized GLM	18.98	25.24	0.75
**Nowcast**	Pre	GT added	Regularized GLM	20.78	25.24	0.82
**Nowcast**	Pre	CET added	Regularized GLM	21.12	25.24	0.84
**Nowcast**	Pre	Mobility added	Regularized GLM	19.66	25.24	0.78
**1W ahead**	Pre	basic	Regularized GLM	19.71	25.24	0.78
**1W ahead**	Pre	GT added	Regularized GLM	20.61	25.24	0.82
**1W ahead**	Pre	CET added	Regularized GLM	21.03	25.24	0.83
**1W ahead**	Pre	Mobility added	Regularized GLM	19.14	25.24	0.76
**2W ahead**	Pre	basic	Regularized GLM	20.02	25.24	0.79
**2W ahead**	Pre	GT added	Regularized GLM	23.76	25.24	0.94
**2W ahead**	Pre	CET added	Regularized GLM	23.86	25.24	0.95
**2W ahead**	Pre	Mobility added	Regularized GLM	21.93	25.24	0.87
**Hindcast**	Early	basic	Regularized GLM	33.05	23.35	1.42
**Hindcast**	Early	GT added	Regularized GLM	29.32	23.35	1.26
**Hindcast**	Early	CET added	Regularized GLM	28.63	23.35	1.23
**Hindcast**	Early	Mobility added	Regularized GLM	24.35	23.35	1.04
**Nowcast**	Early	basic	Regularized GLM	34.19	23.35	1.46
**Nowcast**	Early	GT added	Regularized GLM	32.42	23.35	1.39
**Nowcast**	Early	CET added	Regularized GLM	31.96	23.35	1.37
**Nowcast**	Early	Mobility added	Regularized GLM	26.82	23.35	1.15
**1W ahead**	Early	basic	Regularized GLM	36.63	23.35	1.57
**1W ahead**	Early	GT added	Regularized GLM	35.75	23.35	1.53
**1W ahead**	Early	CET added	Regularized GLM	35.38	23.35	1.52

**1W ahead**	Early	Mobility added	Regularized GLM	30.55	23.35	1.31
**2W ahead**	Early	basic	Regularized GLM	38.44	23.35	1.65
**2W ahead**	Early	GT added	Regularized GLM	37.46	23.35	1.60
**2W ahead**	Early	CET added	Regularized GLM	37.07	23.35	1.59
**2W ahead**	Early	Mobility added	Regularized GLM	34.32	23.35	1.47
**Hindcast**	Post	basic	Regularized GLM	15.01	8.23	1.82
**Hindcast**	Post	GT added	Regularized GLM	19.94	8.23	2.42
**Hindcast**	Post	CET added	Regularized GLM	15.96	8.23	1.94
**Hindcast**	Post	Mobility added	Regularized GLM	4.96	8.23	0.60
**Nowcast**	Post	basic	Regularized GLM	15.15	8.23	1.84
**Nowcast**	Post	GT added	Regularized GLM	19.70	8.23	2.39
**Nowcast**	Post	CET added	Regularized GLM	15.56	8.23	1.89
**Nowcast**	Post	Mobility added	Regularized GLM	5.24	8.23	0.64
**1W ahead**	Post	basic	Regularized GLM	15.72	8.23	1.91
**1W ahead**	Post	GT added	Regularized GLM	20.49	8.23	2.49
**1W ahead**	Post	CET added	Regularized GLM	15.89	8.23	1.93
**1W ahead**	Post	Mobility added	Regularized GLM	5.45	8.23	0.66
**2W ahead**	Post	basic	Regularized GLM	16.12	8.23	1.96
**2W ahead**	Post	GT added	Regularized GLM	20.99	8.23	2.55
**2W ahead**	Post	CET added	Regularized GLM	16.00	8.23	1.94
**2W ahead**	Post	Mobility added	Regularized GLM	5.64	8.23	0.68
